# BRI is an independent predictor of new-onset kidney stones in a non-diabetic population: a retrospective analysis

**DOI:** 10.3389/fendo.2025.1686183

**Published:** 2025-10-15

**Authors:** Xiaohong Fan, Si Yu, Jing Li, Songbai Lin, Sanxi Ai, Haiting Wu, Yunyun Fei, Yan Qin, Gang Chen, Xuemei Li

**Affiliations:** ^1^ Department of Nephrology, Peking Union Medical College Hospital, Beijing, China; ^2^ Department of Health Medicine, Peking Union Medical College Hospital, Beijing, China

**Keywords:** body roundness index (BRI), kidney stones, obesity, prediction, non-diabetic

## Abstract

**Background:**

Kidney stones are a prevalent global health concern with significant morbidity and costs. The body roundness index (BRI), reflecting central fat distribution, might offer improved risk assessment than traditional predictors like body mass index (BMI). This study aimed to determine whether BRI is an independent predictor of new-onset kidney stones in a Chinese cohort and to compare its predictive utility with that of BMI.

**Methods:**

A retrospective cohort analysis was conducted using data from 510,778 physical examinations at Peking Union Medical College Hospital from 1994 to 2024. After exclusions, 26,594 individuals with follow-up exceeding five years were included. Demographic, anthropometric, and laboratory data were collected. Cox proportional hazard models were used to assess the associations between BMI/BRI and kidney stone risk, adjusting for confounders. Stratified analyses were performed by diabetes status.

**Results:**

Among 26,594 participants (mean age 41.2 ± 12.6 years, 50.2% male), 462 developed new-onset kidney stones during follow-up. Individuals with new-onset kidney stones had significantly higher BRI (3.52 vs 3.15, p<0.01), BMI (24.64 vs 23.74 kg/m^2^, p<0.01), and prevalence of metabolic abnormalities (e.g., hypertension, dyslipidemia, hyperuricemia, impaired glucose metabolism; all p<0.01). In unadjusted analysis, both BMI (HR 1.07, 95% CI 1.04-1.09) and BRI (HR 1.29, 95% CI 1.21-1.37) predicted kidney stones. After full adjustment for metabolic confounders, only BRI remained significantly associated with stone risk (adjusted HR 1.13, 95% CI 1.02-1.26), while BMI did not (p=0.86). Stratified analysis revealed that BRI’s predictive value was substantial only in non-diabetic individuals (adjusted HR: 1.17, 95% CI: 1.06–1.30), with no association observed in participants with diabetes (p > 0.05).

**Conclusion:**

BRI, but not BMI, is an independent predictor of new-onset kidney stones in non-diabetic individuals. These findings highlight the importance of visceral adiposity in kidney stone pathogenesis and suggest BRI’s potential utility in risk stratification and preventive strategies.

## Introduction

Kidney stones are a prevalent urological disorder affecting approximately 15% of the global population, with increasing incidence rates attributed to dietary and lifestyle changes (1). The disease poses a significant burden, including recurrent episodes, chronic kidney disease, and substantial healthcare costs (2–4). The occurrence of kidney stone events is associated with a substantially higher risk of end-stage renal disease (5, 6). Early identification of high-risk individuals is critical for implementing preventive strategies; however, current predictive tools remain limited in accuracy and generalizability (7–10). Given the multifactorial etiology of kidney stones, integrating novel biomarkers might help enhance predictive capabilities and aid in patients’ counseling and decision-making.

Traditional predictors of kidney stones include anthropometric measures, such as body mass index (BMI), which have shown inconsistent associations with kidney stone risk across studies (10–15). While BMI reflects general adiposity, it fails to account for visceral fat distribution, a key driver of metabolic dysfunction linked to stone formation (1, 16). Previous studies have indicated that patients with kidney stones have a significantly higher mean visceral fat area (17, 18). In the meantime, the body roundness index (BRI), a geometric index that incorporates waist circumference and height, has demonstrated superior performance in predicting metabolic syndrome and cardiovascular outcomes (19, 20). However, its role in kidney stone prediction remains underexplored, particularly in non-diabetic populations where metabolic dysregulation may independently contribute to kidney stone formation.

This study aims to identify independent predictors of new-onset kidney stones in a large, retrospective cohort. In addition, we aim to investigate whether BRI, as a non-invasive and straightforward measure, can serve as a predictive marker for kidney stone risk in a large population and ultimately inform targeted preventive interventions in at-risk individuals.

## Methods

### Study design and population

**Figure 1:**
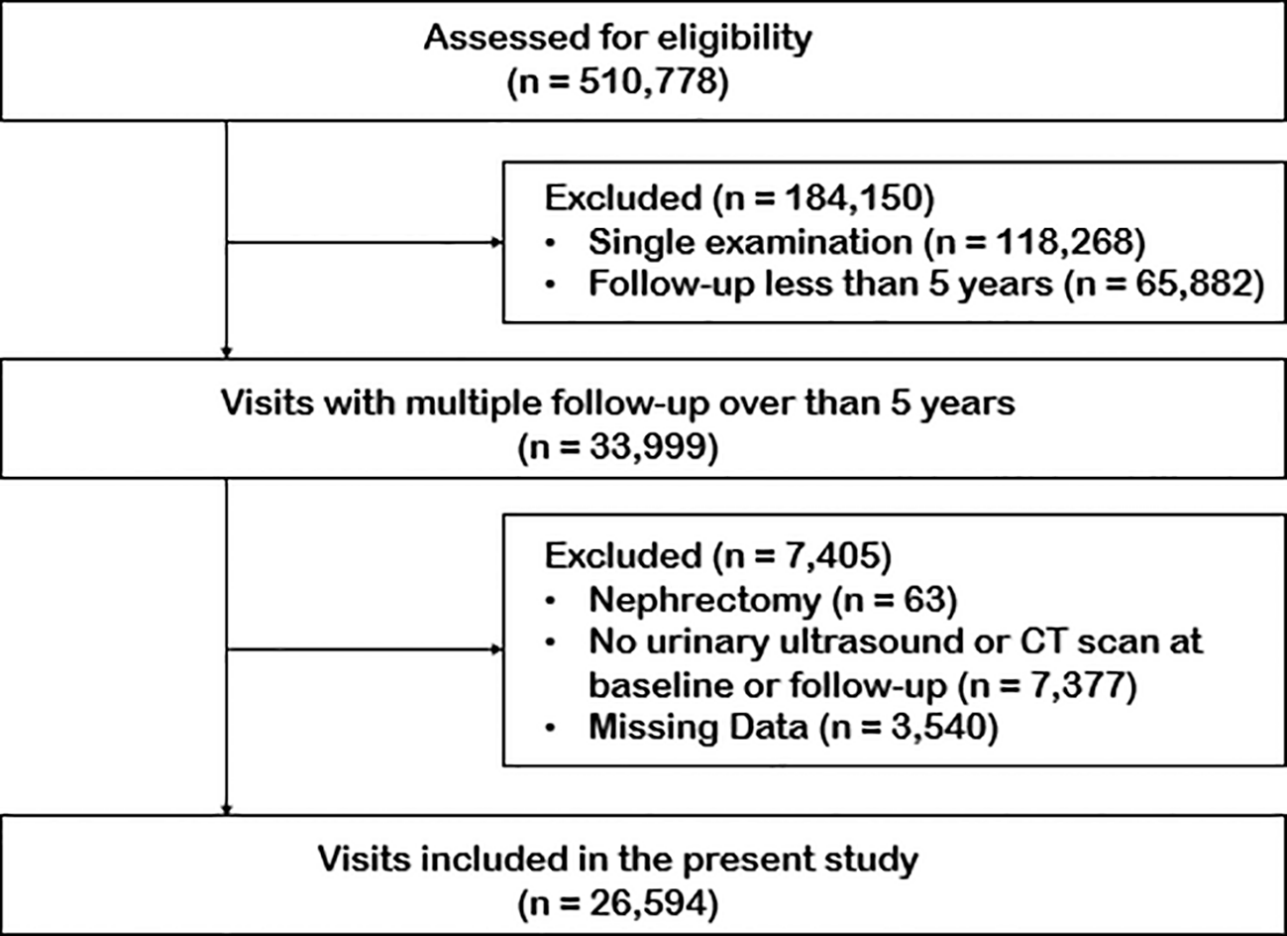
Study flow chart.

The study population was derived from a large dataset of physical examinations in Peking Union Medical College Hospital between 1994 and 2024, encompassing 510,778 visits. From this initial pool, 118,268 visits were identified as single examinations, while 392,510 visits (representing 99,881 individuals) involving multiple follow-ups were included in the following analysis. To ensure the robustness of the analysis, the study focused on individuals with follow-up periods exceeding five years, resulting in a subset of 33,999 participants. Exclusion criteria were applied to refine the cohort: individuals who underwent nephrectomy (n = 63), those without baseline or follow-up urinary ultrasound or CT scans (n = 7,377), and those with missing data (n = 3,540) were excluded. After applying the criteria, the final study cohort comprised 26,594 individuals. The median follow-up time was 71 months (IQR: 61–82 months) (**Figure 1**).

### Study variables and outcome

The study collected a comprehensive set of variables, including demographic and anthropometric data (age, gender, height, weight, waist circumference), blood pressure measurements, laboratory results, and comorbidities. Key calculated variables included:


$$Body\;Mass\;Index\;(BMI)=\frac{Weight(kg)}{Height\;squared(m^{2})}$$

$$Body\;Roundness\;Index\;(BRI)=364.2-365.5\times \sqrt{1-\frac{{\left(\frac{Waist\;circumference[cm]}{2\pi }\right)}^{2}}{{\left(0.5\times Height[cm]\right)}^{2}}}$$

$$Mean\;Blood\;Pressure\;(MBP)=\text{DBP}+\frac{1}{3}(SBP-DBP)$$

$$Triglyceride\;Glucose\;Index\;(TyG\;Index)=ln(\frac{Triglycerides[mg/dL]\times Glucose[mg/dL]}{2})$$

Urine pH was categorized as acidic (<5.5), neutral (5.5–7.0), or alkaline (>7.0). Diabetes and hypertension were defined based on medical history, laboratory criteria (fasting glucose ≥7.0 mmol/L or HbA1c ≥6.5% for diabetes; SBP ≥140 mmHg or DBP ≥90 mmHg for hypertension), and/or current use of relevant medications.

The primary outcome was the development of new-onset kidney stones, identified through urinary ultrasound or computed tomography (CT) scans during follow-ups. All imaging studies were reviewed by board-certified radiologists who were blinded to the exposure variables. The new-onset kidney stone was defined as stones detected during follow-up examinations that were absent on baseline imaging, regardless of symptomatic presentation. Data was collected from medical records and physical examinations using standardized protocols to ensure accuracy and reliability.

### Statistical analysis

Statistical analysis was performed using R (Version 4.2.0). Descriptive statistics were used to summarize the baseline characteristics of the study population. Continuous variables were presented as mean ± standard deviation, and categorical variables were presented as frequencies and percentages. Standardized differences and p-values were calculated to compare the baseline characteristics between individuals with and without new-onset kidney stones. Univariate analysis was performed to assess the association between each variable and the risk of new-onset kidney stones. Collinearity analysis was conducted to identify and exclude variables with high multicollinearity. Potential confounders were defined as variables with a p-value <0.05 in the univariate analysis and variance inflation factors (VIF) less than 5 in the collinearity analysis. To ensure the clinical relevance of the selected variables, we conducted a comprehensive evaluation through collaborative discussions involving experienced clinicians and statistical experts. Cox proportional hazard models were used to assess the associations between BMI/BRI and the risk of new-onset kidney stones. The analysis was performed in three steps: a non-adjusted model, Adjust I (adjusted for gender and age), and Adjust II (further adjusted for the potential confounders). Stratified analysis was performed to examine the association between BRI and new-onset kidney stones in subgroups. We then performed weighted generalized additive models and smoothing curve fitting to assess the role of BRI in the relationship with the new-onset kidney stone risk under diabetic or non-diabetic conditions.

## Results

### Baseline characteristics of the study population

We presented the baseline characteristics of the study population in **Table 1**. The study included 25,783 individuals without kidney stones and 462 with new-onset kidney stones. Participants with new-onset kidney stones were significantly older (47.12 ± 10.18 years vs. 40.74 ± 12.49 years, p<0.01). Their anthropometric measures concerning metabolic status were higher than those without kidney stones, including weight (70.78 ± 12.97 kg vs. 66.95 ± 13.17 kg, p<0.01), waist circumference (85.44 ± 10.49 cm vs. 81.10 ± 10.97 cm, p<0.01), BMI (24.64 ± 3.46 vs. 23.74 ± 3.51, p<0.01), and BRI (3.52 ± 1.07 vs. 3.15 ± 1.07, p<0.01). Additionally, individuals with new-onset kidney stones exhibited higher mean blood pressure (89.14 ± 12.32 mmHg vs. 87.50 ± 11.22 mmHg, p<0.01), higher SBP (118.79 ± 16.40 mmHg vs. 117.01 ± 15.77 mmHg, p=0.017), and DBP (74.32 ± 11.28 mmHg vs. 72.74 ± 10.04 mmHg, p<0.01).

**Table 1:** Baseline characteristics of participants according to incidence of new-onset kidney stone.

Characteristics	None	New-onset kidney stone	Standardized diff.	P-value
N	25783	462		
Age	40.74 ± 12.49	47.12 ± 10.18	0.56 (0.47, 0.65)	<0.01
Height	167.45 ± 8.53	169.08 ± 8.20	0.20 (0.10, 0.29)	<0.01
Weight	66.95 ± 13.17	70.78 ± 12.97	0.29 (0.20, 0.39)	<0.01
Waist circumference	81.10 ± 10.97	85.44 ± 10.49	0.41 (0.31, 0.50)	<0.01
BMI	23.74 ± 3.51	24.64 ± 3.46	0.26 (0.17, 0.35)	<0.01
BRI	3.15 ± 1.07	3.52 ± 1.07	0.35 (0.25, 0.45)	<0.01
MBP	87.50 ± 11.22	89.14 ± 12.32	0.14 (0.05, 0.23)	<0.01
SBP	117.01 ± 15.77	118.79 ± 16.40	0.11 (0.02, 0.20)	0.017
DBP	72.74 ± 10.04	74.32 ± 11.28	0.15 (0.06, 0.24)	<0.1
Glucose	5.35 ± 0.95	5.53 ± 1.37	0.16 (0.06, 0.25)	<0.01
HbA1C	5.47 ± 0.62	5.62 ± 0.81	0.21 (0.09, 0.32)	<0.01
Alkaline phosphatase	61.57 ± 18.12	63.00 ± 16.70	0.08 (-0.01, 0.18)	0.103
Calcium	2.39 ± 0.09	2.37 ± 0.09	0.21 (0.12, 0.31)	<0.01
Phosphate	1.16 ± 0.15	1.12 ± 0.14	0.26 (0.16, 0.35)	<0.01
Total carbon dioxide	24.66 ± 2.27	24.27 ± 2.29	0.17 (0.03, 0.31)	0.014
Uric acid	321.91 ± 86.39	341.80 ± 98.50	0.21 (0.12, 0.31)	<0.01
Total cholesterol	4.76 ± 0.89	4.86 ± 0.88	0.11 (0.02, 0.20)	0.019
Triglyceride	1.33 ± 1.11	1.64 ± 1.35	0.26 (0.16, 0.35)	<0.01
HDL	1.42 ± 0.38	1.31 ± 0.35	0.29 (0.20, 0.38)	<0.01
LDL	2.95 ± 0.78	3.07 ± 0.78	0.15 (0.06, 0.24)	<0.01
TC/HDL ratio	3.59 ± 1.18	3.95 ± 1.27	0.29 (0.20, 0.38)	<0.01
TyG index	8.45 ± 0.61	8.65 ± 0.69	0.31 (0.21, 0.40)	<0.01
Creatinine	73.41 ± 16.02	76.03 ± 15.69	0.17 (0.07, 0.26)	<0.01
Urea	4.55 ± 1.16	4.75 ± 1.16	0.17 (0.08, 0.27)	<0.01
eGFR	101.56 ± 14.93	96.98 ± 14.27	0.31 (0.22, 0.41)	<0.01
C-reactive protein	1.19 ± 2.63	1.08 ± 1.35	0.05 (-0.06, 0.16)	0.442
Gender			0.33 (0.24, 0.42)	<0.01
Female	12854 (49.85%)	156 (33.77%)		
Male	12929 (50.15%)	306 (66.23%)		
Urine pH			0.14 (0.05, 0.24)	<0.01
Acidic	6137 (24.45%)	98 (21.63%)		
Neutral	17272 (68.82%)	307 (67.77%)		
Alkaline	1689 (6.73%)	48 (10.60%)		
Diabetes			0.23 (0.14, 0.32)	<0.01
No	24634 (95.54%)	414 (89.61%)		
Yes	1149 (4.46%)	48 (10.39%)		
Hypertension			0.23 (0.14, 0.32)	<0.01
No	21358 (84.13%)	343 (74.89%)		
Yes	4030 (15.87%)	115 (25.11%)		

BMI, Body mass index; BRI, Body roundness index; MBP, Mean blood pressure; SBP, Systolic blood pressure; DBP, Diastolic blood pressure; HbA1c, Hemoglobin A1c; HDL, High-density lipoprotein; LDL, Low-density lipoprotein; TC, Total cholesterol; TyG, Triglyceride glucose; eGFR, Estimated glomerular filtration rate.

Laboratory findings revealed that participants with new-onset kidney stones had higher levels of glucose (5.53 ± 1.37 mmol/L vs. 5.35 ± 0.95 mmol/L, p<0.01), hemoglobin A1C (5.62 ± 0.81% vs. 5.47 ± 0.62%, p<0.01), uric acid (341.80 ± 98.50 µmol/L vs. 321.91 ± 86.39 µmol/L, p<0.01), triglycerides (1.64 ± 1.35 mmol/L vs. 1.33 ± 1.11 mmol/L, p<0.01), and low-density lipoprotein (3.07 ± 0.78 mmol/L vs. 2.95 ± 0.78 mmol/L, p<0.01), but lower levels of high-density lipoprotein (1.31 ± 0.35 mmol/L vs. 1.42 ± 0.38 mmol/L, p<0.01). Furthermore, individuals with new-onset kidney stones had a higher prevalence of diabetes (10.39% vs. 4.46%, p<0.01) and hypertension (25.11% vs. 15.87%, p<0.01) compared to those without kidney stones.

### Risk factors for new-onset kidney stones

We performed univariate analysis to assess the association between various factors and the risk of new-onset kidney stones and summarized the results in **Supplementary Table 1**. Multiple demographic, anthropometric, clinical, and laboratory factors were significantly associated with the risk of new-onset kidney stones. Among the demographic and anthropometric characteristics, gender, age, height, weight, waist circumference, BMI, and BRI were positively associated with kidney stone risk (all p<0.01). Specifically, BRI is strongly associated with an increased risk of kidney stones (HR 1.29, 95% CI 1.21–1.37, p < 0.01).

### BRI is the independent predictor of new-onset kidney stone

BMI and BRI were selected for further analysis. Variables with high variance inflation factors (VIF > 5), including TC/HDL ratio and TyG index, were excluded from the multivariate Cox proportional hazards models (**Table 2**). In the non-adjusted model, both BMI (hazard ratio (HR) 1.07, 95% confidence interval (CI) 1.04–1.09, p<0.01) and BRI (HR 1.29, 95% CI 1.21–1.37, p<0.01) showed significant positive associations with kidney stone risk. However, after adjusting for gender and age (Adjust I), the association for BMI became non-significant (HR 1.01, 95% CI 0.98–1.04, p=0.489), while BRI remained significant (HR 1.15, 95% CI 1.05–1.26, p<0.01). Only BRI showed a significant association (HR 1.13, 95% CI 1.02–1.26, p=0.019) when further adjusted for additional covariates (Adjust II).

**Table 2:** Association between BMI/BRI and the risks of new-onset kidney stone.

Exposure	N	Models	Per SD increase	P-value
BMI	25744	Non-adjusted	1.07 (1.04, 1.09)	<0.01
	25744	Adjust I^*^	1.01 (0.98, 1.04)	0.489
	23290	Adjust II^**^	1.00 (0.96, 1.03)	0.860
BRI	18507	Non-adjusted	1.29 (1.21, 1.37)	<0.01
	18507	Adjust I^*^	1.15 (1.05, 1.26)	<0.01
	16625	Adjust II^**^	1.13 (1.02, 1.26)	0.019

BMI, Body mass index; BRI, Body roundness index.

*Adjust I model adjust for: gender, age; **The fully adjusted model (Adjust II) included the following covariates: age, gender, mean blood pressure, alkaline phosphatase, calcium, phosphate, uric acid, triglycerides, high-density lipoprotein, low-density lipoprotein, eGFR, urine pH, hypertension, and diabetes status.

### Stratified analysis

**Figure 2:**
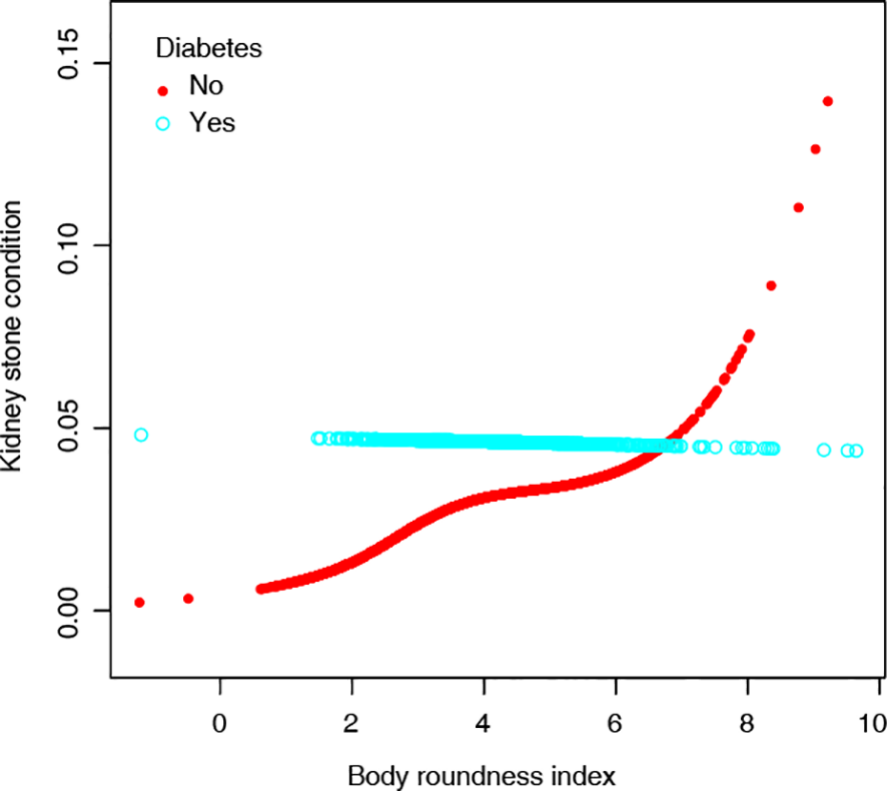
Association between BRI and the risks of New-onset kidney stones in diabetic and non-diabetic populations.

We conducted a stratified analysis to evaluate the association between BRI and the risk of new-onset kidney stones across various subgroups (**Supplementary Table 2**). BRI remained a significant predictor across various subgroups including both genders. Distinguishingly, diabetes status significantly modified the association between BRI and the risk of new-onset kidney stones. In non-diabetic individuals, BRI showed a strong positive association with kidney stone risk in the non-adjusted model (HR 1.28, 95% CI 1.20–1.37, p < 0.01). This association remained significant after adjusting for gender and age (Adjust I: HR 1.16, 95% CI 1.05–1.28, p<0.01) and further adjusting for additional covariates (Adjust II: HR 1.17, 95% CI 1.06–1.30, p<0.01). In contrast, no significant association was observed in diabetic individuals across all models (non-adjusted: HR 1.04, 95% CI 0.80–1.33, p=0.789; Adjust I: HR 0.97, 95% CI 0.75–1.27, p=0.84; Adjust II: HR 0.82, 95% CI 0.60–1.12, p=0.217, **Table 3**). These results suggest that diabetes may overshadow the predictive role of BRI for kidney stone development. Further analysis in the non-diabetic population revealed a gradual increase in kidney stone risk with higher BRI values, with a particularly notable threshold effect observed at BRI > 6. The association between BRI and kidney stone risk remained relatively stable across the BRI range of 4-6 (**Figure 2**).

**Table 3:** Association between BRI and the risks of New-onset kidney stone in diabetic and non-diabetic populations.

Exposure	Models	Per SD increase	P-value
Non-diabetic	Non-adjusted	1.28 (1.20, 1.37)	<0.01
	Adjust I^*^	1.16 (1.05, 1.28)	<0.01
	Adjust II^**^	1.17 (1.06, 1.30)	<0.01
Diabetic	Non-adjusted	1.04 (0.80, 1.33)	0.789
	Adjust I^*^	0.97 (0.75, 1.27)	0.840
	Adjust II^**^	0.82 (0.60, 1.12)	0.217

*Adjust I model adjust for: gender, age; **The fully adjusted model (Adjust II) included the following covariates: age, gender, mean blood pressure, alkaline phosphatase, calcium, phosphate, uric acid, triglycerides, high-density lipoprotein, low-density lipoprotein, eGFR, urine pH, and hypertension status.

## Discussion

Kidney stone is a growing global health concern, with many modifiable factors, including lifestyle, obesity, smoking, and type 2 diabetes, contributing to the development (9, 10). Early identification of high-risk individuals is crucial for implementing preventive strategies. We utilized data from a standardized health examination database to examine the predictors of new-onset kidney stones. Our findings demonstrated that BRI, but not BMI, was significantly associated with kidney stone risk even after adjusting for multiple confounders, highlighting its potential as an anthropometric predictor in clinical practice.

Our study contributes to the growing body of evidence suggesting that traditional measures, such as BMI, may not fully capture the metabolic risks associated with kidney stones. Studies using data from the National Health and Nutrition Examination Survey (NHANES) similarly found that the weight-adjusted waist index and BRI outperformed BMI in association with kidney stones in cross-sectional studies (21–24), aligning with our observation in the Chinese cohort that central adiposity indices, such as BRI, may be more clinically relevant. Specifically, we stratified our analysis by diabetes status, revealing that BRI’s predictive value was significant, especially in non-diabetic individuals. This discrepancy may stem from the dominant metabolic disturbances in diabetic patients, which could overshadow the contribution of adiposity measures.

Our findings underscore the importance of incorporating BRI into kidney stone risk assessments, particularly in non-diabetic populations. Visceral adiposity, as reflected by BRI, is associated with insulin resistance, chronic inflammation, and adipokine production. These metabolic disturbances lead to altered urinary composition, including lower urinary pH, hypercalciuria, hyperoxaluria, and reduced citrate excretion—all established risk factors for calcium oxalate stone formation (25, 26). This mechanistic framework supports why BRI may be a more sensitive predictor of stone risk compared to BMI. Therefore, clinicians should consider waist circumference-based indices in addition to traditional risk factors when evaluating stone risk. Notably, while direct measures of abdominal fat (e.g., ultrasound, CT/MRI) provide precise quantification of visceral adiposity, BRI offers a clinically feasible alternative that leverages routine health examination parameters (waist circumference and height), thereby eliminating the need for specialized imaging and additional costs. These results also underscore the importance of targeting modifiable factors, such as obesity and central adiposity, in kidney stone prevention strategies.

This study has several limitations. First, its retrospective nature may introduce residual confounders despite rigorous adjustments. Second, the study population was derived from a single medical center; the generalizability of the findings needs further validation. Third, due to relatively limited data availability, we could not adjust for confounders including antidiabetic medications and dietary factors in this study, which might potentially neutralize the risk of kidney stones (27); dietary pattern could confound the relationship between BRI and stone risk, as BRI may correlate with diets high in sodium and animal protein, which are known risk factors for stone formation. Future research should prospectively validate BRI’s predictive utility in diverse populations and explore interactions between adiposity indices, antidiabetic medications, dietary habits, and metabolic profiles. Additionally, mechanistic studies investigating how visceral fat contributes to the development of kidney stones might provide deeper biological insights.

In conclusion, our study identifies BRI as an independent predictor of new-onset kidney stones in non-diabetic individuals, offering a more precise tool than BMI for risk stratification. These findings underscore the significance of metabolic health in preventing kidney stones and highlight the need for further research to refine predictive models. By integrating BRI into clinical practice and prioritizing modifiable risk factors, healthcare providers may improve early detection and prevention of this increasingly prevalent condition.

## Data Availability

The raw data supporting the conclusions of this article will be made available by the authors, without undue reservation.

## References

[1] Khan SR Pearle MS Robertson WG Gambaro G Canales BK Doizi S. Kidney stones. Nat Rev Dis primers. (2016) 2:16008. doi: 10.1038/nrdp.2016.8, PMID: 27188687 PMC5685519

[2] Thongprayoon C Krambeck AE Rule AD. Determining the true burden of kidney stone disease. Nat Rev Nephrology. (2020) 16:736–46. doi: 10.1038/s41581-020-0320-7, PMID: 32753740

[3] Uribarri J. Chronic kidney disease and kidney stones. Curr Opin Nephrol hypertension. (2020) 29:237–42. doi: 10.1097/MNH.0000000000000582, PMID: 31972597

[4] Sanz-Gómez I Angerri O Baboudjian M Kanashiro A Gracia S Millán F. Role, cost, and availably of urinary pH monitoring for kidney stone disease-A systematic review of the literature. Curr Urol Rep. (2023) 24:381–8. doi: 10.1111/obr.13023, PMID: 37314611

[5] Alexander RT Hemmelgarn BR Wiebe N Bello A Morgan C Samuel S. Kidney stones and kidney function loss: a cohort study. BMJ (Clinical Res ed). (2012) 345:e5287. doi: 10.1136/bmj.e5287, PMID: 22936784 PMC3431443

[6] Dhondup T Kittanamongkolchai W Vaughan LE Mehta RA Chhina JK Enders FT. Risk of ESRD and mortality in kidney and bladder stone formers. Am J Kidney diseases: Off J Natl Kidney Foundation. (2018) 72:790–7. doi: 10.1053/j.ajkd.2018.06.012, PMID: 30146423 PMC6252145

[7] Garbens A Pearle MS. Causes and prevention of kidney stones: separating myth from fact. BJU Int. (2021) 128:661–6. doi: 10.1111/bju.15532, PMID: 34192414

[8] Micali S Sighinolfi MC Iseppi A Morini E Calcagnile T Benedetti M. Initial experience and evaluation of a nomogram for outcome prediction in management of medium-sized (1–2 cm) kidney stones. Eur Urol focus. (2022) 8:276–82. doi: 10.1016/j.euf.2020.12.012, PMID: 33419709

[9] Vasanthi P Srinivasu LN Teju V Sowmya KV Stan A Sita V. Multiple kidney stones prediction with efficient RT-DETR model. Comput Biol Med. (2025) 190:110023. doi: 10.1016/j.compbiomed.2025.110023, PMID: 40107024

[10] Ma Y Cheng C Jian Z Wen J Xiang L Li H. Risk factors for nephrolithiasis formation: an umbrella review. Int J Surg. (2024) 110:5733–44. doi: 10.1097/JS9.0000000000001719, PMID: 38814276 PMC11392093

[11] Liu M Wu J Gao M Li Y Xia W Zhang Y. Lifestyle factors, serum parameters, metabolic comorbidities, and the risk of kidney stones: a Mendelian randomization study. Front endocrinology. (2023) 14:1240171. doi: 10.3389/fendo.2023.1240171, PMID: 37810889 PMC10560039

[12] Wei B Tan W He S Yang S Gu C Wang S. Association between drinking status and risk of kidney stones among United States adults: NHANES 2007-2018. BMC Public Health. (2024) 24:820. doi: 10.1186/s12889-024-18307-1, PMID: 38491490 PMC10941453

[13] Wang JH Bao EH Chen GY Liu Y Yang L Wang JJ. A positive association between BMI and kidney stones among the diabetic population: a cross-sectional study from NHANES. World J urology. (2024) 42:142. doi: 10.1007/s00345-024-04861-1, PMID: 38478086

[14] Wang Z Lu B Zhang L Tang F Pan Y Zhong S. Association between the atherogenic index of plasma and kidney stones: a nationally representative study. BMC urology. (2024) 24:179. doi: 10.1186/s12894-024-01567-9, PMID: 39182034 PMC11344440

[15] Lovegrove CE Bešević J Wiberg A Lacey B Littlejohns TJ Allen NE. Central adiposity increases risk of kidney stone disease through effects on serum calcium concentrations. J Am Soc Nephrology: JASN. (2023) 34:1991–2011. doi: 10.1681/ASN.0000000000000238, PMID: 37787550 PMC10703081

[16] Shastri S Patel J Sambandam KK Lederer ED. Kidney stone pathophysiology, evaluation and management: core curriculum 2023. Am J Kidney diseases: Off J Natl Kidney Foundation. (2023) 82:617–34. doi: 10.1053/j.ajkd.2023.03.017, PMID: 37565942 PMC11370273

[17] Zhou T Watts K Agalliu I DiVito J Hoenig DM. Effects of visceral fat area and other metabolic parameters on stone composition in patients undergoing percutaneous nephrolithotomy. J urology. (2013) 190:1416–20. doi: 10.1016/j.juro.2013.05.016, PMID: 23685097

[18] Du YZ Yang JQ Tang J Zhang CT Liu YF. Association between the skeletal muscle-to-visceral fat ratio and kidney stones: a cross-sectional study. Front Nutr. (2025) 12:1549047. doi: 10.3389/fnut.2025.1549047, PMID: 40416386 PMC12101122

[19] Rico-Martín S Calderón-García JF Sánchez-Rey P Franco-Antonio C Martínez Alvarez M Sánchez Muñoz-Torrero JF. Effectiveness of body roundness index in predicting metabolic syndrome: A systematic review and meta-analysis. Obes reviews: an Off J Int Assoc Study Obes. (2020) 21:e13023., PMID: 32267621 10.1111/obr.13023

[20] Thomas DM Bredlau C Bosy-Westphal A Mueller M Shen W Gallagher D. Relationships between body roundness with body fat and visceral adipose tissue emerging from a new geometrical model. Obes (Silver Spring Md). (2013) 21:2264–71. doi: 10.1002/oby.20408, PMID: 23519954 PMC3692604

[21] Chen D Xie Y Luo Q Fan W Liu G. Association between weight-adjusted waist index and kidney stones: a propensity score matching study. Front Endocrinol. (2024) 15:1266761. doi: 10.3389/fendo.2024.1266761, PMID: 38911038 PMC11193331

[22] Lin G Zhan F Ren W Pan Y Wei W. Association between novel anthropometric indices and prevalence of kidney stones in US adults. World J Urol. (2023) 41(11):3105–3111. doi: 10.1007/s00345-023-04582-x, PMID: 37716933

[23] Lv G Li X Zhou X Wang Y Gu Y Yang X. Predictive ability of novel and traditional anthropometric measurement indices for kidney stone disease: a cross-sectional study. World J Urol. (2024) 42(1):339. doi: 10.1007/s00345-024-05035-9, PMID: 38767720

[24] Hu X Li X Ye N Zhou Z Li G Jiang F. Association of novel anthropometric indices with prevalence of kidney stone disease: a population-based cross-sectional study. Eur J Med Res. (2024) 29(1):204. doi: 10.1186/s40001-024-01743-5, PMID: 38539239 PMC10967179

[25] Bargagli M Scoglio M Howles SA Fuster DG. Kidney stone disease: risk factors, pathophysiology and management. Nat Rev Nephrol. (2025). doi: 10.1038/s41581-025-00990-x, PMID: 40790363

[26] Ferraro PM Taylor EN Curhan GC. 24-hour urinary chemistries and kidney stone risk. Am J Kidney diseases: Off J Natl Kidney Foundation. (2024) 84:164–9. doi: 10.1053/j.ajkd.2024.02.010, PMID: 38583757 PMC13170619

[27] Anderegg MA Schietzel S Bargagli M Bally L Faller N Moor MB. Empagliflozin in nondiabetic individuals with calcium and uric acid kidney stones: a randomized phase 2 trial. Nat Med. (2025) 31:286–93. doi: 10.1038/s41591-024-03330-x, PMID: 39747681 PMC11750721

